# Evaluation of fracture resistance in class II tooth cavity using different techniques

**DOI:** 10.6026/97320630018858

**Published:** 2022-10-31

**Authors:** Vibhuti Verma, Abhishek Bansal, Navneet Kukreja, Sunpreet Kaur, Prabhu Varsha, Supriya R Zanjad

**Affiliations:** 1 Department of Conservative Dentistry and Endodontics, Maharishi Markendeswar College of Dental Sciences & Research, Mullana, Ambala, Haryana, India; 2 Department of Conservative Dentistry and Endodontics, Saraswati Dental College, Lucknow, UP, India; 3 Department of Conservative Dentistry and Endodontics, Aditya Dental College, Beed, Maharastra, India

**Keywords:** Class II cavity, endodontic, post core, restorative materials, teeth

## Abstract

It is of interest to evaluate the fracture resistance of endodontically and non-endodontically treated teeth with class II cavity using different techniques and materials. Endodontic access cavities were prepared with the diamond fissure bur following the MOD cavity preparations,. The root canals preparation was done followed by obturation using the single cone obturation technique. Later all the samples were embedded in acrylic resin blocks and divided into 8 groups; Group 1: -Intact teeth (Control), Group 2: - MOD (unfilled), Group 3: - MOD restored with composite resin (3M FILTEK P-60 packable), Group 4: - MOD restored with Cention N (Ivoclar Vivadent), Group 5: - 10 mm fiber–post with composite resin, Group 6: - 5mm fiber post with composite resin, Group 7: - Ribbond on the occlusal and composite resin, and Group 8: - Horizontal fiber post with composite resin. Later all samples were subjected for fracture resistance testing using Universal Testing Machine. The mean fracture resistance of Control (513.2 N), Unfilled (60.2N), composite resin (221 N), cention N (88.9 N), 10 mm fiber post (271.4 N), 5 mm fiber post (245 N), Ribbond (456.4N), and horizontal fiber post (338.1N) was found. The fracture resistance values are statistically significant between each group. Best fracture resistance was found to be of intact teeth followed by ribbond on the occlusal surface after endodontic treatment and least fracture resistance of MOD unfilled. Thus, ribbond method is good for the occlusal of teeth compared to other materials for fracture resistance.

## Background:

Tooth decay is one of the most widespread persistent diseases today [1]. As the tooth decay progresses through enamel, dentin and results into pulp pathology, if left untreated. Various restorative materials and techniques are practices to restore the carious teeth and endodontically treated teeth [2]. The root canal preparation can make the tooth structure weaken and hence are more prone to fracture [3]. It has been stated that the primary reason for the frequent fractures of endodontically treated teeth is the loss of structural integrity [4]. Coronal leakage or coronal microleakages are also the contributing factors for the failure of endodontic treatment [5]. For a successful endodontic treatment adequate coronal seal and structural integrity play a very decisive role. Many different restorative materials and methods were introduced for major coronal loss. The different restorative materials suggested for the restoration of endodontically treated and untreated teeth were amalgam, glass ionomer cement, composite resin, and Cention N [5]. There are various types of composites namely, micro fill composite; hybrid composites, micro hybrid composites, packable and flowable composites. With the advancements in adhesive dentistry, the posterior composite resin was very popular [6]. Endodontically treated teeth can be restored with post and core. The main purpose of post-endodontic restorations in endodontically treated teeth is to provide lost resistance to the occlusal masticatory load [7]. Retention of the post is also directly related to the length of the post, i.e., retention will be higher when a post will be placed deeply [8]. A post is advised only when there is extensive damage to the tooth structure, including coronal structure loss; which needs some form of retention for the core [9]. Class II cavities (MOD) represent the common clinical conditions which weaken the tooth structure the most, leading to a decrease in rigidity of the tooth by 63% [10]. Due to ultra-high-modulus of elasticity and better adhesion to synthetic restorative materials after treating them with cold gas plasma including light or chemically cured composite resins, polyethylene fiber (RIBBOND- a reinforced ribbon) became quite popular as they increased the fracture strength of prosthetic, orthodontic and restorative materials [11,12]. Glass fiber-reinforced epoxy resin posts were used in this study, because of their esthetic looks. They became popular due to the high demand for all-ceramic restorations. The glass fiber reinforced posts comprise white or translucent glass or silica fibers. Three different types of glasses can be used for their making - electrical glass, high-strength glass, or quartz fibers [13]. The resin cement used to cement post provides adequate sealing between the root dentin wall and the surface of the post [14]. Ribbond is the alternative material being available to provide strength greater or equal to fiber post. Due to ultra-high-modulus of elasticity and better adhesion to synthetic restorative materials after treating them with cold gas plasma with composite resins, polyethylene fiber (RIBBOND- a reinforced ribbon) became quite popular as they increased the fracture strength of prosthetic, orthodontic and restorative materials [11,12]. Cention N is a new tooth-colored alkaline resin-based restorative material that can be self-cured having a higher flexural strength as compared with composite. Cention -N is considered as a modification in tooth-colored restorative materials after composite. It is a resin-based, self-cured like an ormocer or compomer with "alkaline filler" with an ability to neutralize the acidic ions [5]. It acts as a remineralizing agent to enamel due to the release of ample amount of calcium and fluoride ions [10]. Therefore, it is of interest to evaluate the fracture resistance of endodontically and non-endodontically treated teeth with class II cavity using different techniques and materials.

## Material and Methodology:

This in vitro study was done in the Department of Conservative Dentistry and Endodontics in Maharishi Markendeshwar College of Dental Sciences & Research, Mullana, Ambala. A total of 80 extracted mandibular premolars were divided into 8 groups (n=10). All collected teeth were disinfected with thymol and cleaned with ultrasonic cleaner and stored in a jar containing distilled water until further use. Using a high-speed diamond fissure bur (MANI Inc., India) underwater cooling, standardized MOD cavities were prepared with 4.5mm width and extending 2mm below CEJ (cementoenamel junction) with a remaining dentin thickness of 2 ± 0.5 mm (for all of the teeth, except the control group (intact teeth). Following the MOD cavity preparations, endodontic access cavities were prepared with the diamond fissure bur under water cooling in group 5-8. The root canals preparation was done using WaveOne Gold (Dentsply- large files 45.5%) followed by obturation using the single cone obturation technique using AH Plus sealer. In groups with vertical post of varying length, post space was prepared using Piezo reamer (number 2) and luted with Calibra self-adhesive resin cement (Dentsply). Later all the samples were embedded in acrylic resin blocks (2 cm in width and 3 cm in height) to simulate clinical conditions in a way that only the crown portion was visible. This acrylic resin block were divided into 8 groups based on the approach of the coronal restoration being followed as: Group 1: -Intact teeth (Control), Group 2: - MOD (unfilled), Group 3: - MOD restored with composite resin (3M FILTEK P-60 packable , Group 4: - MOD restored with Cention N (Ivoclar Vivadent , Group 5: - 10 mm fiber –post with composite resin, Group 6: - 5mm fiber post with composite resin, Group 7: - Ribbond on the occlusal and composite resin (a groove was made of 1mm depth on occlusal surface and ribbond was first placed in bonding agent for few minutes and then placed in this groove, and Group 8: - Horizontal fiber post with composite resin .In this group using round diamond bur with air-water cooling, holes were prepared at the center of both buccal and lingual cusps of premolars, and fiber post was inserted in a horizontal direction and luted using flowable composite. Later all samples were subjected for testing fracture resistance using Universal Testing Machineat an angle of 45° obliquely. A compressive load was applied at the buccal cusp of restored teeth at the junction of enamel tissue and composite filling with a crosshead pace of 0.5 mm/min till a fracture occurred. The maximum load before fracture was recorded in kilograms which were then converted into Newton.

### Statistical analysis:

The collected data were analyzed using SPSS (IBM Corporation Software Group, 19.0 Version) using One-way ANOVA for collective differentiation and Post-hoc Tukey HSD test for comparison between two groups.

## Results:

The mean fracture resistance of all 8 groups i.e., Control (513.2 N), Unfilled MOD (60.2N), composite resin (221 N), cention N (88.9 N), 10 mm fiber post (271.4 N), 5 mm fiber post (245 N), Ribbond (456.4N), and horizontal fiber post (338.1N). The fracture resistance values are statistically significant between each group. Table 1(see PDF) indicates the comparison of fracture resistance among groups. The results revealed that group 1 (intact), group 7 (Ribbond on occlusal & composite resin), group 8 (Horizontal fiber post & composite resin) were found to be statistically significantly better than group 5 (5mm fiber post & composite resin), group 6 (10mm fiber post & composite resin), group 3 (MOD with composite resin), group 4 (MOD with Cention- N) and group 2 (MOD unfilled). The result was statically significant between the groups (P<0.01). Group 1 > Group 7 > Group 8 > Group 5 > Group 6 > Group 3 > Group 4 > Group 2. Best fracture resistance was found to be in intact teeth followed by ribbond on the occlusal surface after endodontic treatment and least fracture resistance of MOD unfilled.

## Discussion:

Over the past 25 years, Conservative Dentistry and Endodontics have seen advancement in the form of new instruments, techniques, and materials used for root canal therapy [15]. By virtue of this strong technological expansion, endodontic therapy allows the clinical practitioners to effectively manage teeth with endodontic complications [16]. This study evaluated the fracture resistance of endodontically and non-endodontically treated teeth with class II cavities using different techniques and materials. The Universal Testing Machine is designed such that at low velocity and consequently high resolution, it produces small deformations. Karzoun et al. determined fracture resistance after root canal treatment for group1 as intact (control), group 2 as (MOD unfilled), group 3 (MOD with composite resin), group 4 (MOD with horizontal fiber post & composite resin), while group 5 (MOD with horizontal fiber post alone). They concluded that mean fracture resistance of group 1(intact-control) has the higher as compared with group 2(MOD unfilled) [17]. In contrast to our result, Karzoun et al. stated that mean fracture resistance values that group composite resinrestoration has the higher fracture resistance as compared with group unfilled [17]. Chowdhury et al. conducted a study to check the fracture resistance of various restoration materials in class II cavity including amalgam, Z350 composite resin, and cention-N. They concluded that cention N and Z350 composite resin can strengthen the tooth structure more efficiently [10]. Sound tooth has the highest fracture resistance because of the presence of intact tooth structure available [17]. While decrease in fracture resistance of tooth with Class II cavity because marginal ridge reduces the remaining dentin available and hence least fracture resistance without any reinforcing restorative material [18]. Bahari et al. assessed the influence of various fibers on composite resin strength after the root canal treatment in intact, no restoration, and composite, fibers on the occlusal surface, horizontal post, Bucco-palatally and occlusal fibers horizontal fiber post Bucco-palatally methods. They concluded that composite has higher fracture resistance when compared with MOD unfilled one. This was because composite restored the fracture resistance of the tooth similar to the intact tooth while there was no reinforcing material in case of MOD unfilled [19]. Mishra et al. compared the strength of Cention N with amalgam, GIC, and hybrid composite. They concluded that composite had better strength than Cention N [20]. It was found that, Cention N has lesser marginal integrity when compared with composite [12]. Schwartz et al. suggested that the minimum post length required is 8mm.Longer posts tend to absorb more stresses due to their larger capacity (mass), rather than transferring it to radicular dentin [21]. Bahari et al. stated that the use of ribbond in root canal treated teeth with a MOD cavity has a greater fracture resistance as they can transmit forces equally at the interface of dentin and restoration [19]. Belli et al. stated by saying that ribbond has a better fracture resistance due to its high young's modulus and less bending modulus at tooth and restoration junction [22]. Ribbond helps to transfer the forces uniformly throughout the junction of resin and restoration leading to numerous paths of stress in fiber [3]. Bahari et al. found an increase in fracture resistance when fiber post is being placed horizontally [19]. The limitations of the present study were; forces generated by a machine can't stimulate the intra-oral masticatory forces, pace, amplitude, and direction of intra-oral forces. However, further research is needed with more studies and a greater number of samples with various materials; techniques and considering various other factors.

## Conclusions:

It was concluded that intact tooth (control) has the highest fracture resistance followed by ribbond on the occlusal surface of endodontically treated tooth with composite resin and horizontal fiber post and composite resin.

## References

[1] Kishen A (2006). Endodontic Topics..

[2] Mincik J (2016). Int J Biomat..

[3] Sagsen B, Aslan B (2006). International Endodontic Journal..

[4] Hervas Garcia A (2006). Med Oral Patol Oral Cir Bucal..

[6] Kiremitci AR (2009). Operative dentistry..

[7] Stockton LW (1999). The Journal of prosthetic dentistry..

[8] Franco EB (2014). The Journal of Prosthetic Dentistry..

[9] Morgano SM (1996). J Prosthet Dent.

[11] Aslan T (2018). Niger J Clin Pract..

[12] Karaman AI (2002). American Journal of Orthodontics and Dentofacial Orthopedics..

[15] Koch M (2014). Intl Endodontic J..

[16] Strindberg LZ (1956). The dependence of the results of pulp therapy on certain factors. An analytic study based on radiographic and clinical follow-up examinations. Doctoral Thesis,.

[17] Karzoun W (2015). Journal of Endodontics..

[18] Cobankara FK (2008). Operative dentistry..

[19] Bahari M (2019). Pesquisa Brasileiraem Odontopediatria e Clinica Integrada..

[21] Schwartz RS, Robbins JW (2004). Journal of endodontics..

[22] Belli S (2005). International Endodontic Journal..

